# Transplantation rénale entre époux et immunisation anti-HLA en prégreffe: à propos de deux observations

**DOI:** 10.11604/pamj.2021.40.92.18744

**Published:** 2021-10-12

**Authors:** Asmaa Drissi Bourhanbour, Sanae Ouadghiri, Sara Bougar, Ouafae Atouf, Chahrazade Brick, Imane Yakhlef, Kaoutar Atiifis, Kaoutar El Morabit, Malika Essakalli

**Affiliations:** 1 Service d'Immunologie et de Transfusion, Centre Hospitalier Universitaire Ibn Sina de Rabat, Rabat, Maroc,; 2 Faculté de Médecine et de Pharmacie, Université de Hassan II, Casablanca, Maroc,; 3 UPR d'Immunologie, Faculté de Médecine et de Pharmacie, Université Mohamed V de Rabat, Rabat, Maroc

**Keywords:** Immunisation, époux, greffe, rein, rapport de cas, Immunization, husband, transplant, kidney, case report

## Abstract

La transplantation rénale est la meilleure thérapeutique pour l'insuffisance rénale terminale. Cette transplantation est possible grâce au don de rein à partir d'un donneur vivant ou d'un donneur en état de mort encéphalique (EME). L'immunisation des receveurs est une vraie problématique de la greffe car elle est responsable de difficultés particulières de choix d'un donneur et surtout expose au risque de rejet de greffon. Nous allons présenter deux observations de greffe rénale entre époux, ou les deux receveurs avaient des taux faibles d'anticorps dirigés contre des antigènes HLA du donneur mais dont l'issue en post-greffe immédiat était différente selon le sexe du receveur. En effet l'immunisation anti-HLA des femmes suite aux grossesses est un vrai obstacle à leur greffe par le rein de leur époux. Malgré la faible compatibilité HLA qui caractérise la transplantation rénale entre les époux, car le donneur est ici non apparenté, cette transplantation offre une bonne alternative aux greffes de reins à partir de donneurs en EME, qui font cruellement défaut au Maroc.

## Abstract (en)

Renal transplantation is the best therapeutic approach for end-stage kidney disease. Renal transplantation can be performed using living donors or brain-dead donors. Vaccination in recipients poses a real problem with the transplantation process because it is responsible for particular difficulties in choosing a donor and above all exposes to the risk of transplant rejection. We here report two cases of husband to wife renal transplantation. The recipients had low levels of antibodies against HLA antigens of donors but sex-associated differences in post-transplant results were found. Indeed, HLA immunization after pregnancy is a real obstacle to husband to wife renal transplantation. Despite the low husband-wife HLA matching (unrelated donor), renal transplantation is a good alternative for renal transplantation from brain-dead donors, who are lacking in Morocco.

## Introduction

Le nombre de patients en insuffisance rénale chroniques terminales (IRCT) est en constante augmentation dans le monde. Au Maroc, l'incidence de l'IRCT est de 60 par million d'habitants, avec plus de 10 632 patients hémodialysés [1]. La transplantation rénale est la meilleure thérapeutique dans ce cas. En effet, le rein transplanté permet le plus souvent d'assurer normalement toutes les fonctions du rein, d'augmenter la survie des patients et assurer aussi une meilleure qualité de vie. La greffe rénale est possible grâce au don de rein à partir d'un donneur vivant ou d'un donneur en état de mort encéphalique (EME). La législation marocaine autorise le don vivant à partir des ascendants, des descendants, des frères, des sœurs, des oncles, des tantes du donneur ou de leurs enfants mais aussi à partir du conjoint à condition que le mariage soit contracté depuis une année au moins.

La transplantation rénale nécessite la réalisation d'un bilan immunologique avant la greffe. Ce bilan comporte le typage HLA classe I et II pour permettre le meilleur appareillement entre le donneur et le receveur. La recherche des anticorps anti-HLA permet de mettre en évidence la présence d'éventuels anticorps dans le sérum du receveur qui seraient dirigés contre les antigènes HLA du donneur *(Donor Specific Antibody; DSA)*. Ces anticorps apparaissent suite à une immunisation par des grossesses, des transfusions ou des greffes et sont délétères pour le greffon et exposent au risque de rejet humoral [2]. La recherche des anticorps anti-HLA comprend une étape de dépistage suivi d'une étape d'identification des spécificités d'anticorps en cas de positivité. La recherche de ces anticorps se fait par plusieurs technologies innovantes (La cytométrie en flux ou la technologie Luminex). La technologie Luminex est la plus sensible. C'est une technique de fluorimétrie en flux utilisant des microbilles de polystyrène recouvertes d'antigènes HLA purifiés. Un cross match lymphocytaire sera réalisé pour juger de la compatibilité finale par la mise en présence des lymphocytes du donneur et le sérum du receveur, ce dernier test permet d'éviter un rejet hyper aigu. L'immunisation des receveurs est un obstacle majeur à la greffe car elle est responsable de difficultés particulières de choix d'un donneur et surtout expose au risque de rejet de greffon.

Dans cet article nous allons présenter et discuter deux observations de greffe rénale entre époux, ou les deux receveurs avaient des taux faibles de DSA mais dont l'issue en post-greffe immédiat était différente.

## Patient et observation

### Observation n°1

**Présentation du patient:** il s'agit d'un homme âgé de 60 ans, en IRC et hémodialysé depuis 20 ans sur néphropathie lithiasique. L'anamnèse retrouve des antécédents de transfusions en 1997 et 2000 par quatre culots globulaires au total. Ce patient est candidat à une greffe par le rein de son épouse.

**Figure 1:**
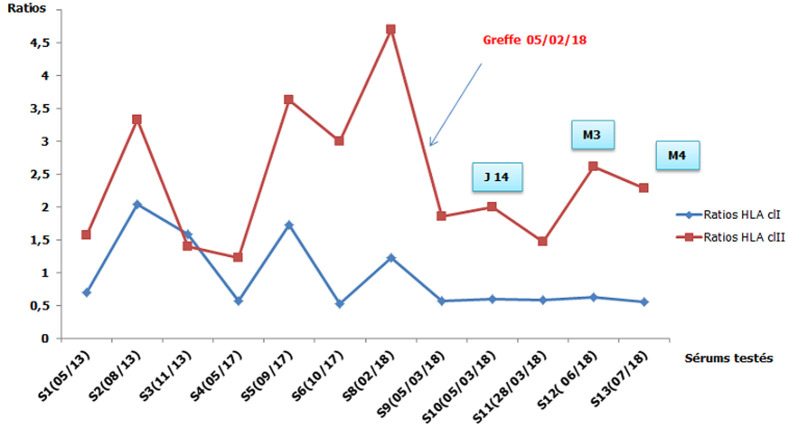
cinétique des anticorps anti-HLA en pré et post greffe du patient de la première observation (*J14: quatorze jours après la greffe, M3: trois mois après la greffe, M4: quatre mois après la greffe*)

**Figure 2:**
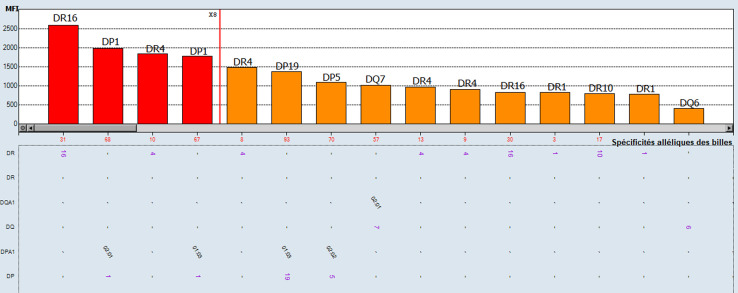
anticorps anti-HLA classe II identifiés en pré-greffe par *Single antigen* première observation

**Démarche diagnostique:** les typages HLA du donneur et du receveur montrent l'existence de deux identités HLA en classe I; A29, B44 (Tableau 1). La recherche des anticorps anti-HLA a été faite par la technologie Luminex avec le réactif One Lambda™. Le dépistage des anticorps anti-HLA sur 7 sérums testés en pré-greffe était négatif en classe I et positif en classe II. Dans notre laboratoire, le seuil de positivité pour le dépistage des anticorps anti-HLA est un ratio supérieur à 3 (Figure 1). Devant la positivité du dépistage, une identification des anticorps anti-HLA classe II a été réalisée et elle a mis en évidence la présence d'anticorps anti-HLA non spécifique du donneur (non DSA) qui atteignaient 2500 de moyenne de fluorescence (MFI) (Figure 2). Deux DSA ont été détectés à des taux inférieur au seuil de positivité déterminé au laboratoire par 1000 de MFI. Ces DSA sont; l'anti-DR1 à 970 de MFI et l'anti-DQ6 à 454 de MFI. Le cross match lymphocytaire par la technique de lymphocytotoxicité dépendante du complément était négatif.

**Tableau 1:** données immunologiques des deux couples de greffe rénale

	Observation 1	Observation 2
**Typage HLA receveur**	**A29** A68 **B44** B51	A1 A28 **B14** B17
	DRB1*03 DRB1*07	DRB1*11 DRB1*13
	DQB1*02 DQB1*02	DQB1*03 DQB1*05
**Typage HLA donneur**	**A29** A33 **B44** B65	A11 A26 **B14** B52
	DRB1*01 DRB1*13	DRB1*01 DRB1*15
	DQB1*05 DQB1*06	DQB1*05 DQB1*06
**Lien de parenté du donneur**	Epouse	Epoux
**Evénement immunisant**	Transfusions	Grossesse et transfusion

**Intervention thérapeutique:** au terme de ce bilan, la greffe a été autorisée et le patient a été greffé avec succès. Le patient a reçu un traitement d'induction par l'antithymoglobuline (ATG) et les corticoïdes puis un traitement d'entretien par le tacrolimus et le mycophénolatemofétil (MMF).

**Suivi et résultats:** le dépistage des anticorps anti-HLA en post-greffe était négatif en classe I et positif en classe II sur six sérums (Figure 1). L'identification des anticorps anti-HLA classe II a montré une diminution des taux des DSA détectée avant la greffe (Tableau 2). Actuellement et à une année après la greffe, le rein est fonctionnel avec un taux des anticorps anti-HLA stationnaire.

**Tableau 2:** évolution des MFI des DSA en pré et post greffe du premier patient

	MFI des DSA	
DSA	Anti-DR1	Anti-DQ6
**S5 pré-greffe**	506	-
**S8 pré-greffe**	970	454
**S13 post-greffe (M4)**	717	239

S: sérum; DSA: anticorps spécifique du donneur; MFI: intensité moyenne de fluorescence

### Observation n°2

**Présentation du patient:** il s'agit d'une femme âgée de 33 ans, en IRC suite à une néphropathie indéterminée, candidat à une greffe par le rein de son époux. Elle a deux événements immunisants; une transfusion et une grossesse en 2008.

**Figure 3:**
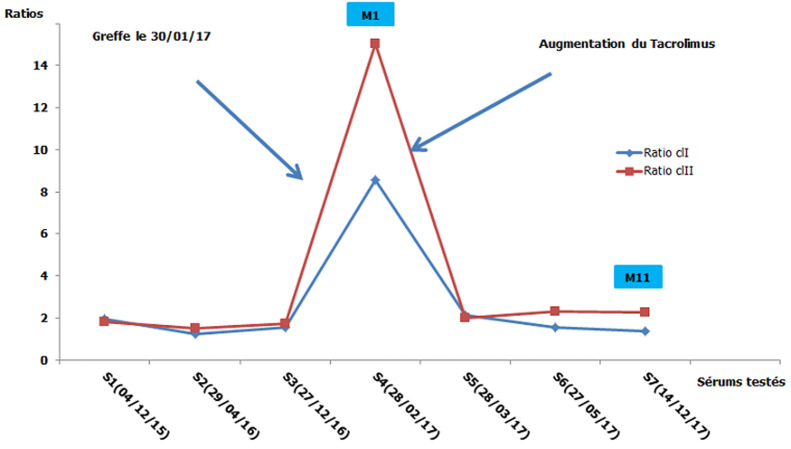
cinétique des anticorps anti-HLA en pré et post greffe du patient de la deuxième observation (*M1: un mois après la greffe, M10: dix mois après la greffe*)

**Figure 4:**
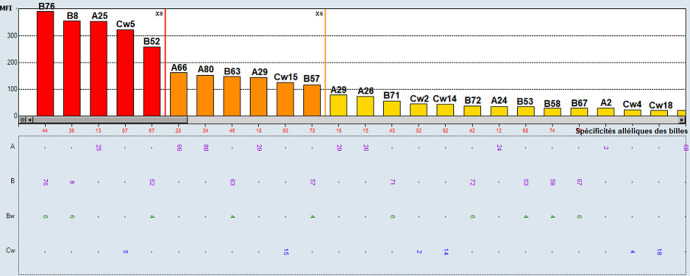
anticorps anti-HLA classe I identifiés en pré-greffe par Singel antigen du patient de la deuxième observation;

**Figure 5:**
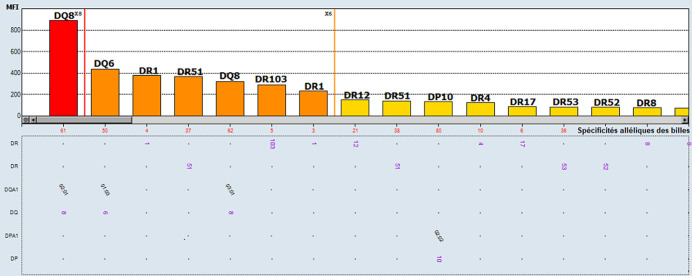
anticorps anti-HLA classe II identifiés en pré-greffe par Singel antigen du patient de la deuxième observation

**Démarche diagnostique:** les typages HLA du donneur et du receveur montrent l'existence de deux identités HLA; B14 et DQB1*06 (Tableau 1). La recherche des anticorps anti-HLA a été faite par la technologie Luminex avec le réactif One Lambda^TM^. Le dépistage des anticorps anti-HLA sur 3 sérums testés en pré-greffe étaient négatives en classe I et II (Figure 3). Une identification des anticorps anti-HLA a été réalisée vu les antécédents d'immunisation et elle a mis en évidence la présence d'anticorps anti-HLA non DSA et plusieurs DSA à des taux inférieurs au seuil de positivité (Figure 4, Figure 5). Ces DSA sont au nombre de cinq: l'anti-A26 à 237,83 de MFI, l'anti-B52 à 389,65 MFI, l'anti-DR1 à 389,24 MFI, l'anti-DR15 à 139,41 et l'anti-DQ6 à 505,99 MFI. Le cross match lymphocytaire par la technique de lymphocytotoxicité dépendante du complément était négatif.

**Intervention thérapeutique:** la patiente a été greffée avec succès. Elle a reçu le protocole suivant; un traitement d'induction par Tacrolimus et les corticoïdes puis un traitement d'entretien qui comportait les corticoïdes, le Tacrolimus et le MMF.

**Suivi et résultats:** le dépistage des anticorps anti-HLA à j30 en post-greffe était positif avec des ratios de 8 pour la classe I et de 15 pour la classe II. L'identification de ce sérum a montré une augmentation du taux des DSA détectés en pré-greffe (Tableau 3) et l'apparition d'un DSA de novo à un seuil inférieur à 1000 de MFI. Devant ce tableau; les cliniciens ont augmenté la dose du Tacrolimus pour éviter le rejet. Un an après la greffe, le rein est toujours fonctionnel avec une recherche des anticorps anti-HLA négative.

**Tableau 3:** évolution des MFI des DSA en pré et post greffe du deuxième patient

	MFI des DSA					
DSA	Anti-A11	Anti-A26	Anti-B52	Anti-DR1	Anti-DR15	Anti-DQ6
**S1 pré-greffe**	-	237.83	389.65	389.24	139.41	505.99
**S5 post-greffe (M1)**	637.12	313.08	3390.57	400.95	3381.87	5380.93

S: sérum; DSA: anticorps spécifique du donneur; MFI: intensité moyenne de fluorescence

## Discussion

L'immunisation anti-HLA demeure un problème important en transplantation rénale. Pour tous les candidats à la greffe rénale, il est nécessaire de réaliser une recherche d'anticorps tous les trois mois en l'absence d'immunisation et à j15 et j30 suivant un événement immunisant pour détecter d'éventuels anticorps anti-HLA. Dans la littérature, la transfusion entraîne une immunisation anti-HLA chez environ 40% des patients transfusés [3]. L'antécédent de transfusion est retrouvé chez le receveur de rein de la 1^re^ observation et qui s'est immunisé contre les antigènes HLA de classe II. Parmi les anticorps HLA identifiés, il y avait deux anticorps dirigés contre les antigènes HLA de son épouse. La receveuse de rein de la 2^e^ observation avait en plus de la transfusion la notion de grossesse. Plusieurs études ont montré que 50% des femmes développent des anticorps anti-HLA après trois grossesses. Cette immunisation n'est pas toujours stable dans le temps, elle peut ne plus être détectable quelques années après la grossesse et être “réactivée” par un nouveau stimulus antigénique [4]. Ces anticorps sont dirigés contre les antigènes HLA du mari et que les enfants ont hérité.

Dans notre cas, la receveuse a développé des anticorps anti-HLA dirigé contre cinq antigènes HLA du mari. La greffe par le rein de son époux a entrainé une réactivation du système immunitaire et une augmentation des taux des DSA en post-greffe avec apparition d'un DSA de novo. Chez le receveur de rein de la 1^re^ observation le taux des DSA a diminué après la greffe pas le rein de son épouse. Dans ce cas, la formation des DSA était due aux antigènes HLA portés probablement par les plaquettes des culots globulaires transfusés et non par les antigènes HLA propre à l'épouse.

L'immunisation anti-HLA des femmes suite aux grossesses est un vrai obstacle à leur greffe par le rein de leur époux. En effet, le sexe féminin du receveur a une influence significativement négative sur le devenir de la greffe. Ceci s'explique par la présence d'une immunisation de la receveuse vis-à-vis des antigènes HLA de son donneur, lorsqu'il s'agit de son conjoint ou d'un de ses enfants [5].

Des études sur la transplantation rénale entre conjoints, ont montré que la survie du greffon est superposable à celle de la meilleure des transplantations cadavériques [6, 7]. D'autres études ont rapporté que le taux de survie du greffon est le même dans le cas de greffe entre époux et le cas de greffe à partir de donneur vivant apparenté [8, 9]. Ces résultats doivent être interprétés avec prudence car probablement ces études n'incluaient pas les femmes immunisées contre leurs maris et qui étaient exclues d'emblée de la greffe rénale.

Les DSA sont associés à un risque accru de rejet humoral et impactent la survie du greffon. Selon des études, la survie du greffon ne serait impactée qu'en présence d'un DSA préexistant avec une valeur de MFI > 3000 [10]. Chez nos deux receveurs, les DSA étaient à des taux faibles de MFI (< 1000). D'autres études rapportent l'effet du cumul des MFI dans le rejet de greffe [11]. Chez nos deux receveurs le cumul des MFI des DSA était inférieur au seuil critique de 3000 de MFI (1424 de MFI et 1662 de MFI respectivement).

## Conclusion

En conclusion malgré la faible compatibilité HLA qui caractérise la transplantation rénale entre les époux, car le donneur est ici non apparenté, cette transplantation offre une bonne alternative aux greffes de reins à partir de donneurs en EME, qui font cruellement défaut au Maroc. En effet, le bénéfice d'un greffon signifie pour nos patients une transplantation qui dure longtemps et dans de bonnes conditions de fonction rénale même si parfois l'immunisation anti-HLA des femmes suite aux grossesses est un vrai obstacle à leur greffe par le rein de leur époux.
